# Oxidative stress markers-driven prognostic model to predict post-discharge mortality in heart failure with reduced ejection fraction

**DOI:** 10.3389/fcvm.2022.1017673

**Published:** 2022-11-07

**Authors:** Imen Gtif, Rania Abdelhedi, Wael Ouarda, Fériel Bouzid, Salma Charfeddine, Fatma Zouari, Leila Abid, Ahmed Rebai, Najla Kharrat

**Affiliations:** ^1^Laboratory of Screening Cellular and Molecular Process, Centre of Biotechnology of Sfax, University of Sfax, Sfax, Tunisia; ^2^Digital Research Center of Sfax, University of Sfax, Sfax, Tunisia; ^3^Unit of Cardiology in Hospital of Hedi Chaker, Faculty of Medicine, University of Sfax, Sfax, Tunisia

**Keywords:** heart failure, oxidative stress, mortality, prediction, models

## Abstract

**Background:**

Current predictive models based on biomarkers reflective of different pathways of heart failure with reduced ejection fraction (HFrEF) pathogenesis constitute a useful tool for predicting death risk among HFrEF patients. The purpose of the study was to develop a new predictive model for post-discharge mortality risk among HFrEF patients, based on a combination of clinical patients’ characteristics, N-terminal pro-B-type Natriuretic peptide (NT-proBNP) and oxidative stress markers as a potentially valuable tool for routine clinical practice.

**Methods:**

116 patients with stable HFrEF were recruited in a prospective single-center study. Plasma levels of NT-proBNP and oxidative stress markers [superoxide dismutase (SOD), glutathione peroxidase (GPX), uric acid (UA), total bilirubin (TB), gamma-glutamyl transferase (GGT) and total antioxidant capacity (TAC)] were measured in the stable predischarge condition. Generalized linear model (GLM), random forest and extreme gradient boosting models were developed to predict post-discharge mortality risk using clinical and laboratory data. Through comprehensive evaluation, the most performant model was selected.

**Results:**

During a median follow-up of 525 days (7–930), 33 (28%) patients died. Among the three created models, the GLM presented the best performance for post-discharge death prediction in HFrEF. The predictors included in the GLM model were age, female sex, beta blockers, NT-proBNP, left ventricular ejection fraction (LVEF), TAC levels, admission systolic blood pressure (SBP), angiotensin-converting enzyme inhibitors/angiotensin receptor II blockers (ACEI/ARBs) and UA levels. Our model had a good discriminatory power for post-discharge mortality [The area under the curve (AUC) = 74.5%]. Based on the retained model, an online calculator was developed to allow the identification of patients with heightened post-discharge death risk.

**Conclusion:**

In conclusion, we created a new and simple tool that may allow the identification of patients at heightened post-discharge mortality risk and could assist the treatment decision-making.

## Introduction

Heart failure (HF) is a complex clinical syndrome resulting from any functional or structural heart disorder, leading to a reduction of cardiac output or an increase in intracardiac pressures (1). HF is a major clinical and public health concern that affects around 63.4 million people worldwide, accounting for an economic burden of 346.17 billion US $ worldwide in 2017 (2–4). For African countries, HF is still health challenging and was associated with significant rates of hospitalizations and mortality (5–8).

Based on the measurement of the left ventricular ejection fraction (LVEF), HF with reduced ejection fraction (HFrEF) and HF with preserved ejection fraction (HFpEF) are the two major HF subtypes (9). HFrEF is a progressive and multifactorial disease, mainly associated with left ventricular systolic dysfunction and adverse cardiac remodeling (10). It develops as the final and serious stage of various cardiac diseases, including coronary artery disease, myocarditis, valve disease, arterial hypertension and arrhythmias (11). Although the significant advance in HF management, HFrEF remains a serious public health problem with high morbidity, hospitalizations and mortality rates (12–14). A study combining the Cardiovascular Health Study and Framingham Heart Study cohorts reported that 67% of HFrEF patients died within 5 years after diagnosis (15). Thus, prediction of mortality risk for HFrEF patients becomes essential to guide therapy decision-making. Indeed, several demographic characteristics, comorbidities, clinical variables and HF medications have been identified as relevant predictors of mortality among patients with HFrEF (16–18). The Natriuretic peptides, including B-type Natriuretic peptide (BNP) and N-terminal pro-B-type Natriuretic peptide (NT-proBNP), are the gold standard biomarkers used in diagnosis, risk stratification and prediction of future cardiac events in HFrEF patients (19–21). Furthermore, the measurement of specific biomarkers, associated with the different pathways of HFrEF pathogenesis has emerged as the most appropriate approach to facilitate the prediction of mortality risk in patients with HFrEF (19, 22–26).

An ever-growing body of evidence supports that increased oxidative stress, resulting from an imbalance between the production of reactive oxygen species (ROS) and antioxidant defense mechanisms, is involved in the pathogenesis of HFrEF (27, 28). Indeed, increased production of ROS causes cellular dysfunction, protein oxidation, lipid peroxidation, and nucleic acid damage. These alterations contribute to myocyte apoptosis, cardiomyocyte hypertrophy, collagen deposition and matrix remodeling eventually leading to progressive left ventricular remodeling and dysfunction driving HFrEF (29). The components of the antioxidant defense systems, responsible for the inactivation of ROS, consist of antioxidant enzymes such as superoxide dismutase (SOD), catalase, glutathione peroxidase (GPx), peroxiredoxins; non-enzymatic antioxidants, including glutathione (GSH), vitamins, uric acid (UA), total bilirubin (TB) and albumin (30). The assessment of markers relevant to antioxidant defense systems had indicated an association with the progression and severity of HFrEF (31–33). Furthermore, there is growing evidence that antioxidant parameters may provide valuable new insight into the prognosis of HFrEF. Indeed, a large number of studies have been conducted to prove the potential role of UA as a prognostic marker in HFrEF (34–36). The gamma-glutamyl transférase (GGT), the first enzyme of the gamma glutamyl cycle that regulates the antioxidant GSH, has emerged as a promising biomarker for predicting mortality among patients with HFrEF (37, 38). In 2019, Romuk et al. demonstrated that SOD activity was associated with long-term outcomes in HFrEF (39). Other studies showed that total antioxidant capacity (TAC) and bilirubin levels were associated with an increased risk of death in patients with HFrEF (36, 40).

Based on these findings, we hypothesized that oxidative stress markers in combination with NT-proBNP and relevant clinical factors may provide a good predictive potential for mortality risk in HFrEF. Accordingly, the present study aimed to develop a new predictive model for post-discharge mortality risk among HFrEF patients, based on a combination of clinical patient characteristics, NT-proBNP and oxidative stress markers, as a potentially valuable tool for routine clinical practice.

## Materials and methods

### Patients and study design

This study is a prospective single-center study. A total of 116 consecutive patients, admitted for newly diagnosed or exacerbated HFrEF to the Cardiology Department of CHU Hedi Chaker from November 2017 to December 2019, were recruited. This study was approved by the local ethics committee of CHU Hédi Chaker of Sfax (Tunisia), in accordance with the principles expressed in the Declaration of Helsinki (CPP Sud 0276/2017). Written informed consent was obtained from all enrolled patients.

The diagnosis of HFrEF was based on the Framingham criteria and the presence of left ventricular systolic reduction (LVEF < 50%) (41, 42). Only patients discharged alive were evaluated in the present study. The exclusion criteria were: Age < 20 years, HFpEF (LVEF ≥ 50%), acute myocardial infarction, a severe valvular disease requiring surgery, renal failure requiring dialysis, presence of inflammatory disease, autoimmune diseases and malignant diseases.

### Data collection

Patients’ demographic and clinical characteristics, comorbidities and treatments are known to influence the prognosis of HFrEF were documented from medical records and through patient interviews. For each patient, the following characteristics were collected: age sex, comorbidities [hypertension, diabetes mellitus, hyperlipidemia, chronic kidney disease (CKD), chronic obstructive pulmonary disease (COPD) and anemia], HF characteristics [previous history of HF, New York Heart Association (NYHA) class and ischemic etiology], clinical measures [body mass index (BMI), systolic blood pressure (SBP), LVEF, electrocardiogram indicators (atrial fibrillation (AF), left Bundle Branch Block (LBBB) and QRS duration) and creatinine clearance (CC)] and discharge medications [beta blockers, angiotensin-converting enzyme inhibitors/angiotensin receptor II blockers (ACEI/ARBs), loop diuretics, aldosterone antagonist and statins]. The etiology of HFrEF was classified as ischemic or non-ischemic, based on a history of myocardial infarction and/or coronary angiography. LVEF was determined by two-dimensional echocardiography, using the biplane Simpson’s method (43). CC was estimated using the Cockcroft-Gault Equation (44). The prognostic outcome of the present study was post-discharge all-cause mortality. Information regarding outcomes was obtained through hospital records and telephone contact with patients or their close family members. The follow-up time was calculated from discharge to all-cause mortality (time to death) or termination of the study.

### Biochemical measurements

Fasting blood samples were collected under stable conditions before discharge. Samples were centrifuged upon permanent cooling at 3,500 rpm for 5 min. Obtained plasma was stored immediately at −20°C temperature until assay. UA, TB, and GGT were measured using the Hitachi 912 analyzer (Roche).

SOD activity was measured by the method of Beyer and Fridovich (45), based on the ability of SOD to inhibit the oxidation of nitro blue tetrazolium (NBT) in the presence of oxygen. The reduction of NBT was measured by a spectrophotometer at 560 nm. SOD activity was calculated by determining the percentage inhibition per min under standard conditions. A 50% of inhibition corresponds to one unit of SOD activity.

GPx activity was determined according to the method of Flohé and Günzler (46), based on glutathione oxidation by GPx in the presence of Ellman’s Reagent (DTNB). The absorbance was measured at 412 nm. GPx activity was expressed as nmoles of disappeared GSH/min/mg of proteins.

TAC was measured by colorimetric method using the Colorimetric Assay Kit (Catalog #K274-100; BioVisionIncorporated; CA 95,035 USA). According to the manufacturer’s instructions, the antioxidant equivalent concentrations were measured at 570 nm as a function of Trolox concentration. TAC was expressed as mM Trolox equivalent.

NT-proBNP levels were assessed by the Human NT Pro-BNP DuoSet ELISA kit (DY3604-05, R&D, Minneapolis, MN, USA). According to the manufacturer’s protocol, the double-antibody sandwich method was applied in this assay. The measurement range of the NT-proBNP assay was 312–10000 pg/ml.

### Statistical analysis

For descriptive statistics, the Shapiro-Wilk test was used to assess the normality of continuous variables. Continuous variables were presented as mean and standard deviations (SD) or median and interquartile range [(IQR): Q3–Q1] according to their distribution. Categorical variables were expressed as numbers and percentages. To examine the differences in biomarker levels and clinical characteristics between survivors and non-survivors, *T*-tests were used for parametric variables, U Mann–Whitney tests for non-parametric variables and Chi-square tests for categorical variables. The level of statistical significance was set at a two-tailed *p*-values < 0.05.

The association between oxidative stress markers and post-discharge mortality risk was evaluated by Kaplan-Meier (KM) survival analysis, log-rank test and Cox proportional hazards regression. Receiver operating characteristic (ROC) curves were used to determine the relevant cut-off of biomarkers statistically associated with post-discharge mortality for the identification of low-risk and high-risk subjects. KM survival curves were then generated to illustrate survival of patients, according to cut-off values of these biomarkers and Log rank tests were used to compare between the curves. Univariate Cox proportional hazards regression analysis was performed to determine the predictive value of each biomarker and each baseline patient characteristic. Variables with statistical significance in the univariate Cox analysis (*p*-values < 0.05) were then adjusted for age, sex and BMI in a multivariable model. Multivariate analyses were eventually conducted using the backward stepwise selection process. Variables with *p*-values ≤ 0.1 were selected in the multivariate proportional hazards regression analysis. Results are presented as hazard ratios (HR) with a 95% confidence interval (CI).

### Development of post-discharge mortality risk prediction models

We developed three predictive models, including the generalized linear model (GLM), random forest (RF, based on bootstrap model aggregation of classification trees) model and extreme gradient boosting (XGBoost) model. Thirty baseline variables were put into the prognostic models, including demographics, comorbidities, clinical factors, medications and biochemical variables (Table 1). A bi-directional stepwise procedure (backward and forward), method that minimize the akaike information criterion (AIC), was used for GLM variable selection with a significance level at 0.10 as criteria to retain significant variables in the model. The AIC was used to avoid model overfitting. Results are reported as odds ratios (OR) with 95% CI. Shapley additive explanation (SHAP) values were used to evaluate the variables’ importance in the RF and XGBoost models. Leave-One-Out Cross-Validation (LOOCV), a special case of *k*-fold cross validation with *k* equal to *n* (the number of observations in the data set) (47), was applied in order to evaluate models’ performance. The evaluation metrics used in this study were area under the receiver operating characteristic curve (AUC), accuracy, recall, precision, F1-score and Matthews correlation coefficient (MCC). The best performing model was then selected to predict post-discharge mortality risk in this study. Subsequently, we calculated the probability of death using the predictors of the selected model. Finally, patients were classified into high and low risk groups according to this probability and the KM curve was performed for survival analysis. Statistical analysis was performed using SPSS (Statistical Package for the Social Sciences) version 23 and R statistical software version 3.3.3 (R Project for Statistical Computing). Machine learning algorithms were performed using Python version 3.9 (Python Software Foundation) (Supplementary material).

**TABLE 1:** Baseline characteristics of study patients stratified according to prognosis outcome.

		Post-discharge mortality during follow-up		
		
	Total (116)	Yes (33)	No (83)	*P*-value
**Demographics**				
Age, years	62.5 ± 11.6	65.7 ± 12.3	61.3 ± 11.1	0.067
Sex *n* (%)				
Male	81 (72.4)	20 (60.6)	64 (77.1)	0.061
Female	32 (27.6)	13 (39.4)	19 (22.9)	
**Comorbidities**				
Hypertension (Yes) *n* (%)	44 (37.9)	13 (39.4)	31 (37.3)	0.5
Diabetes mellitus (Yes) *n* (%)	33 (28.4)	10 (30.3)	23 (27.3)	0.474
Hyperlipidemia (Yes) *n* (%)	30 (25.9)	10 (30.3)	20 (24.3)	0.321
CKD (Yes) *n* (%)	22 (18.9)	10 (30.3)	12 (14.5)	**0.047**
COPD (Yes) *n* (%)	8 (6.9)	4 (12.12)	4 (4.8)	0.159
Anemia (Yes) *n* (%)	53 (45.7)	18 (54.5)	35 (42.16)	0.158
**Heart failure characteristics**				
Previous history of HF *n* (%)	55 (47.4)	21 (63.6)	34 (40.9)	**0.023**
NYHA class III/IV *n* (%)	83 (71.5)	29 (87.9)	54 (65.1)	**0.010**
LVEF (%)	30 (15–48)	25 (15–45)	30 (15–48)	**0.034**
Ischemic etiology *n* (%)	47 (40.5)	12 (36.4)	35 (42.2)	0.360
**Clinical measures**				
BMI (Kg/m^2^)	25.5 (17.4–36.3)	24.2 (18.8–33.9)	25.9 (17.3–36.3)	0.664
Admission SBP (mm Hg)	120 (77–180)	110 (77–170)	120 (88–180)	**0.003**
QRS duration (ms)	118 (74–196)	108 (80–196)	100 (74–196)	**0.044**
AF *n* (%)	46 (39.6)	13 (39.4)	33 (39.7)	0.571
LBBB *n* (%)	49 (42.2)	18 (54.5)	31 (37.3)	0.069
CC (ml/min)	73.5 (33–174)	60 (33–141)	78 (33–174)	**0.025**
**Discharge medications**				
Beta blockers (yes) *n* (%)	90 (77.6)	21 (63.6)	69 (83.1)	**0.024**
ACEI/ARBs (yes) *n* (%)	62 (53.4)	11 (33.3)	51 (61.4)	**0.006**
Loop diuretics (yes) *n* (%)	100 (86.2)	31 (93.9)	69 (83.1)	0.106
Aldosterone antagonist (yes) *n* (%)	56 (48.3)	14 (42.4)	42 (50.6)	0.278
Statins (yes) *n* (%)	58 (50)	17 (51.5)	41 (49.4)	0.5
**Biochemical variables**				
UA (μmol/l)	429.5 (71–1000)	530 (71–970)	401 (224–1000)	**0.005**
TB (g/l)	15 (4–76)	17 (5–73)	15 (4–76)	0.210
GGT (UI/l)	37 (8.2–197)	36 (14–127)	37 (8.2–197)	0.788
SOD (UI/l)	117.4 (74–174)	120.2 (82.9–174.8)	109.7 (74–174.1)	0.650
GPx (nmol/min/mg protein)	2.6 (1–6.32)	2.5 (1.3–6.3)	2.6 (1–5.7)	0.753
TAC (mM Trolox equivalent)	10.9 ± 1.7	11.4 ± 1.5	10.6 ± 1.7	**0.023**
NT-proBNP (pg/ml)	3550 (354–7,000)	4393.33 (1,140–7,000)	3380 (354–6733.33)	**0.001**

ACEI/ARBs, angiotensin-converting enzyme inhibitors/angiotensin receptor II; AF, atrial fibrillation; BMI, body mass index; CC, creatinine clearance; COPD, chronic obstructive pulmonary disease; CKD, chronic kidney disease; GPx, glutathione peroxidase; HF, heart failure; LBBB, left Bundle Branch Block; LVEF, left ventricular ejection fraction; NT-proBNP, N-terminal pro-B-type Natriuretic peptide; NYHA, New York Heart Association; SBP, systolic blood pressure; SOD, superoxide dismutase; UA, uric acid; TAC, total antioxidant capacity; TB, total bilirubin. Bold values indicate the *p*-values < 0.05.

## Results

### Study population characteristics

#### Clinical patients’ characteristics

A total of 116 patients with HFrEF were followed for 525 days (7–930). Baseline patients’ characteristics and the difference between died patients and those surviving during the follow-up period are summarized in Table 1. Overall, the study patients present a mean age of 62.5 ± 11.6 years and were predominantly male (72%). Indeed, anemia and hypertension were the most prevalent comorbidities among study patients. A total of 55 (47.4%) patients had a previous history of HF and the majorities (71.5%) were in NYHA class III/IV. More than 50% of patients had a non-ischemic etiology for HF. The median LVEF was 30% (15–48). Loop diuretics (86.2%), beta blockers (77.6%) and angiotensin-converting enzyme inhibitors/angiotensin receptor II blockers (ACEI/ARBs) (53.4%) were the most common medications prescribed to patients at hospital discharge (Table 1).

During follow-up period, 33 (28%) patients died. The most frequent cause of death was HF in 60% of cases. Non-survivors were more likely to have CKD (30.3 vs. 14.5%, *p* = 0.047), previous history of HF (63.6 vs. 40.6%, *p* = 0.023) and NYHA class III/IV symptoms (78.9 vs. 65.1%, *p* = 0.010). They also had a lower SBP [110 mm Hg (77–170) vs. 120 mm Hg (88–180), *p* = 0.003], lower LVEF [25% (15–45) vs. 30% (15–48), *p* = 0.034] and lower CC rate [60 ml/min (33–141) vs. 78 ml/min (33–174), *p* = 0.025] compared with survivors. The death group had a higher QRS duration [108 ms (80–196) vs. 100 ms (74–196), *p* = 0.044]. The dead patients were less likely to be treated with ACEI/ARBs (33.3 vs. 61.4%, *p* = 0.006) and beta blockers (63.6 vs. 83.1%, *p* = 0.024).

#### Biochemical parameters

Among study patients, the median plasma concentrations of UA and TB were 429.5 (71–1000) μmol/l and 15 (4–76) g/l, respectively. Median plasma GGT, GPx and SOD activities were 37 (8.2–197) UI/l, 2.6 (1–6.32) to nmol/min/mg protein and 117.4 (74–174) UI/l, respectively. The mean plasma TAC levels were 10.9 ± 1.7 mM Trolox equivalents. The median plasma NT-proBNP levels were 3550 (354–7000) pg/ml. When analyzing oxidative stress marker levels, non-survivors had higher values of UA [530 μmol/l (71–970) vs. 401 μmol/l (224–1000), *p* = 0.005] and TAC (11.4 ± 1.5 mM trolox equivalent vs. 10.6 ± 1.7 mM trolox equivalent, *p* = 0.023). However, GPx, SOD, and GGT activities and TB concentration were not statistically different between the two groups. The death group had also elevated levels of NT-proBNP [4393.33 pg/ml (1140–7000) vs. 3380 pg/ml (354–6733.33), *p* = 0.001].

### Association between oxidative stress markers and the risk of all-cause mortality

#### Kaplan-Meier survival analysis

**FIGURE 1:**
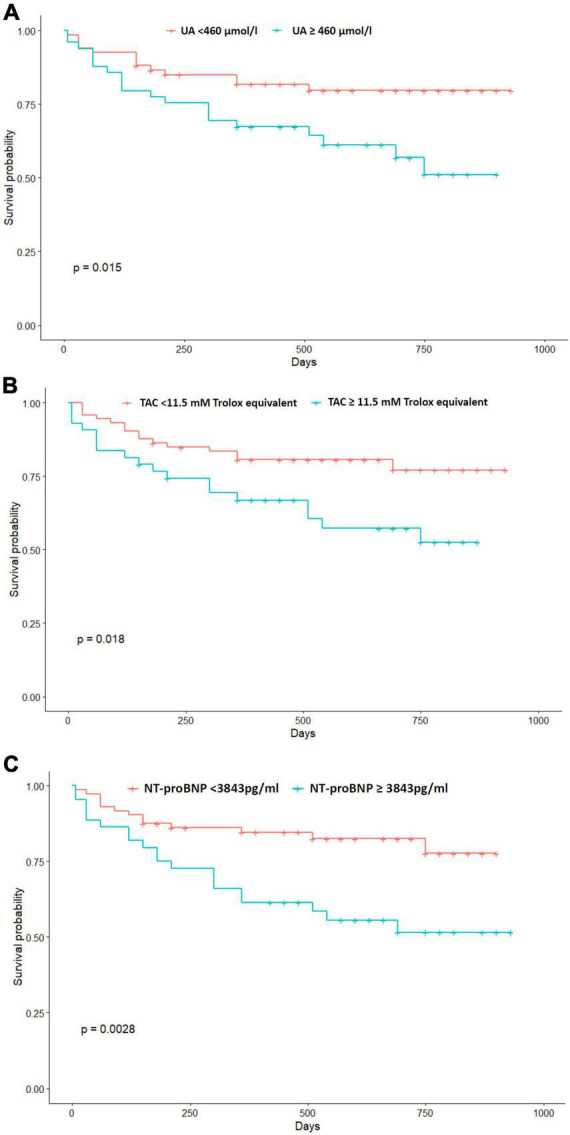
Kaplan-Meier event-free survival curves for post-discharge mortality relative to plasma levels of UA **(A)**, TAC **(B)** and NT-proBNP **(C)** above or below cut-off values.

We performed the KM analysis to estimate survival probabilities for all-cause mortality, according to cut-off values of UA, TAC, and NT-proBNP. The ROC curve analysis showed that the best cut-off value for UA, TAC, and NT-proBNP to predict all-cause mortality risk were 460 μmol/l (60% sensitivity, 65% specificity, 67% AUC), 11.5 mM trolox equivalent (55%sensitivity, 70% specificity, 65% AUC) and 3843 pg/ml (60% sensitivity, 71% specificity, 70% AUC), respectively. KM survival curves illustrate an increasing risk of mortality rate among patients with UA levels above 460 μmol/l (log-rank test *p* = 0.015). Furthermore, subjects with TAC levels above 11.5 mM trolox equivalent were more likely to die during follow-up period (log-rank test *p* = 0.018) (Figure 1). The predictive utility of NT-proBNP levels for death risk among HFrEF patients was also evaluated by KM survival curves. Log-rank test showed that patients with NT-proBNP levels above 3843 pg/ml are more likely to experience death (log-rank test *p* = 0.0028) (Figure 1).

#### Uni and multivariate cox regression analysis

**FIGURE 2:**
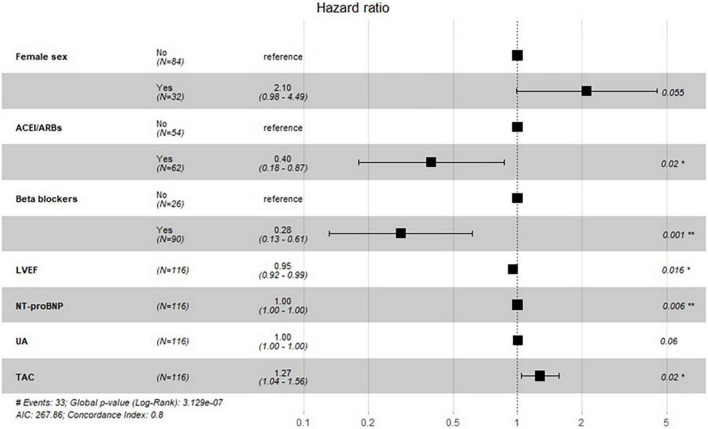
Multivariable Cox regression for post-discharge mortality prediction.

In univariate Cox-regression analysis, highest UA levels (HR 1.002, 95% CI 1.000–1.004, *p* = 0.042) and elevated TAC levels (HR 1.126, 95% CI 1.016–1.478, *p* = 0.033) were significant predictors of post-discharge mortality. Additional significant determinants of mortality risk were revealed in univariate analysis, including: previous history of HF, NYHA class III/IV, NT-proBNP levels, LVEF, admission SBP, QRS duration, CC rate, beta blockers and ACEI/ARBs (Table 2). In order to evaluate the independent association of UA and TAC in the context of other common clinically available data, a multivariate model was performed, including sex, age, BMI and all significant clinical predictors. Stepwise multivariate Cox-regression analysis revealed that elevated UA levels (HR 1.001, 95% CI 1.000–1.003, *p* = 0.06) of high TAC levels (HR 1.272, 95% CI 1.040–1.560, *p* = 0.02) remained independent predictors of death. In the multivariate Cox model, female sex, lower LVEF, and high NT-proBNP levels were also independent predictors for post-discharge mortality. Furthermore, multivariate analysis showed that patients taking of beta blockers or ACEI/ARBs after hospital discharge faced a lower risk of death during the follow-up period (Figure 2).

**TABLE 2:** Univariate Cox proportional hazards regression analysis for predictors of post-discharge mortality.

Variable	HR	95% CI	*P*-value
Age	1.028	0.997–1.016	0.076
Female sex	1.798	0.894–3.616	0.1
Hypertension	1.011	0.503–2.033	0.976
Diabetes mellitus	1.055	0.502–2.219	0.887
Hyperlipidemia	1.283	0.609–2.701	0.520
CKD	1.966	0.948–4.199	0.069
COPD	2.179	0.762–6.235	0.146
Anemia	1.547	0.779–3.072	0.212
Previous history of HF	2.198	1.080–4.471	**0.030**
NYHA class III-IV	3.316	1.165–9.438	**0.025**
LVEF	0.951	0.912–0.991	**0.017**
BMI	0.976	0.890–1.076	0.612
Admission SBP	0.972	0.954–0.990	**0.003**
QRS duration	1.016	1.003–1.029	**0.021**
AF	0.946	0.471–1.903	0.877
LBBB	1.843	0.928–3.658	0.080
CC	0.986	0.972–1.000	**0.045**
ACEI/ARBs	0.359	0.173–743	**0.006**
Beta blockers	0.426	0.209–0.867	**0.019**
Diuretics	2.609	0.624–10.91	0.189
Aldosterone antagonist	0.784	0.393–1.565	0.491
Statins	1.058	0.543–2.096	0.872
UA	1.002	1.000–1.004	**0.042**
TB	1.018	0.997–1.040	0.098
GGT	0.998	0.990–1.007	0.715
GPx	1.046	0.713–1.534	0.819
SOD	1.006	0.991–1.020	0.457
TAC	1.226	1.016–1.478	**0.033**
NT-proBNP	1.001	1.000–1.001	**< 10^–3^**

ACEI/ARBs, angiotensin-converting enzyme inhibitors/angiotensin receptor II; AF, atrial fibrillation; BMI, body mass index; CC, creatinine clearance; COPD, chronic obstructive pulmonary disease; CKD, chronic kidney disease; GPx, glutathione peroxidase; HF, heart failure; LBBB, left Bundle Branch Block; LVEF, left ventricular ejection fraction; NT-proBNP, N-terminal pro-B-type Natriuretic peptide; NYHA, New York Heart Association; SBP, systolic blood pressure; SOD, superoxide dismutase; UA, uric acid; TAC, total antioxidant capacity; TB, total bilirubin. Bold values indicate the *p*-values < 0.05.

### Risk prediction model for post-discharge mortality

**FIGURE 3:**
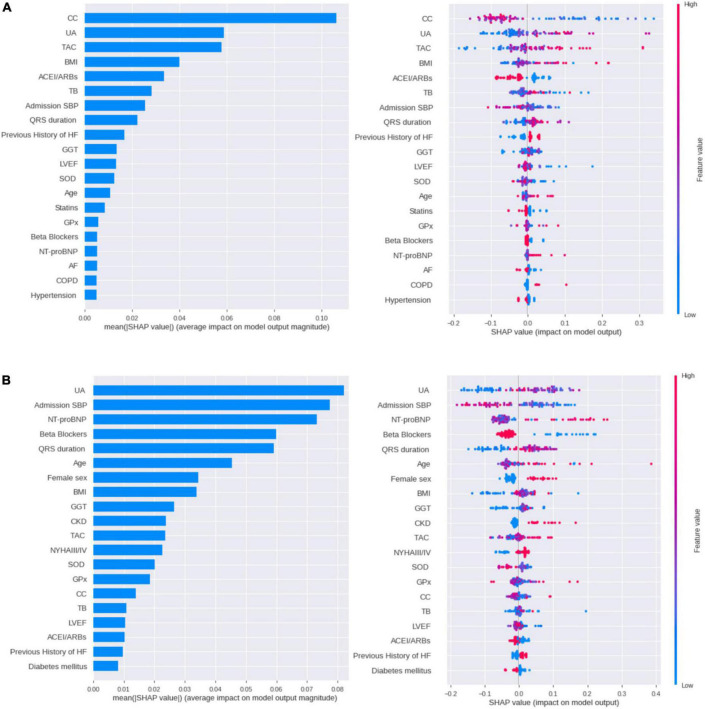
SHAP plots for the ML models in predicting post-discharge mortality using **(A)** RF and **(B)** XGBoost. In each variable importance row, all patients’ attribution to mortality risk was plotted using different color dots. The red dots represent the highest risk of death.

In order to predict the post-discharge mortality risk among HFrEF patients, GLM, RF, and XGBoost models were performed using clinical and laboratory data. Regarding GLM, 9 independent predictors of mortality were retained in the final model. They included beta blocker, NT-proBNP levels, female sex, LVEF, TAC levels, admission SBP, ACEI/ARBs, UA levels and advanced age (Table 3). In the RF and XGBoost models, SHAP values were used to explain how the selected features affect the mortality prediction. In each variable importance row, all patients’ attribution to post-discharge death risk were plotted with dots of different colors where the blue dots represent the lowest risk value and the red dots represent the highest risk value (Figure 3). In RF model, the top 5 related variables in mortality prediction were CC rate, UA levels, TAC levels, BMI and ACEI/ARBs. In XGBoost model, UA levels were the most important identified feature, followed by admission SBP, NT-proBNP levels, beta blockers and QRS duration. Among the three predictive models created, the GLM presented the best performance. This model achieved the highest AUC (74.5%), accuracy (81.9%), recall (58%), precision (65%), F1-score (64%) and MCC (53%) compared to the respective values in the RF and XGboost models (Table 4). Therefore, the GLM model was selected to predict the risk of post-discharge death in the present study. The estimated β-coefficients of Glm-selected variables were used to estimate the logit for a patient using the standard GLM equation. The estimated individual probability (*P*) of dying was then calculated using the following formula:


$$P=\frac{e^{L\,o\,g\,i\,t}}{1+e^{L\,o\,g\,i\,t}}$$

**TABLE 3:** Predictive model for post-discharge mortality based on stepwise generalized linear model.

Variable	β-coefficient	OR (95% CI)	*P*-value
Age	0.054	1.055 (1.002–1.117)	0.048
Female sex	1.480	4.392 (1.304–16.411)	0.020
LVEF	0.778	0.925 (0.859–0.988)	0.027
Admission SBP	–0.022	0.978 (0.947–1.006)	0.142
NT-proBNP	0.001	1.001 (1.0002–1.001)	0.009
UA	0.003	1.003 (1.0001–1.006)	0.044
TAC	0.347	1.414 (1.027–2.009)	0.039
ACEI/ARBs	–1.180	0.307 (0.093–0.949)	0.044
Beta blockers	–1.825	0.161 (0.038–0.596)	0.008

ACEI/ARBs, angiotensin-converting enzyme inhibitors/angiotensin receptor II; LVEF, left ventricular ejection fraction; NT-proBNP, N-terminal pro-B-type Natriuretic peptide; SBP, systolic blood pressure; UA, uric acid; TAC, total antioxidant capacity.

**TABLE 4:** Performance comparison between the three models.

	AUC	Accuracy	Precision	Recall	F1-score	MCC
GLM	74.5%	81.89%	73%	58%	64%	53.1%
RF	63%	75.86%	52%	36%	43%	33.3%
XGBoost	61.5%	72.4%	65%	33%	44%	26.1%

AUC, area under curve; GLM, generalized linear model; MCC, matthews correlation coefficient; RF, random forest; XGBoost, extreme gradient boosting.

**FIGURE 4:**
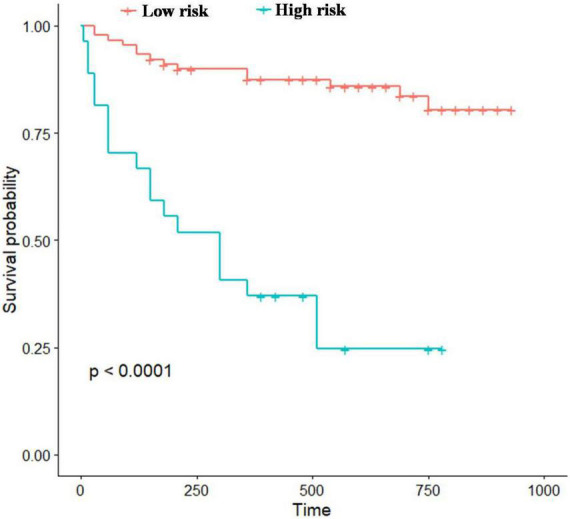
Kaplan–Meier survival analysis between the high-and low-risk groups.

According to the estimated probability, study patients were divided into high (*P* > 0.5) and low (*P* ≤ 0.5) risk groups and KM curve survival analysis was applied. The result of the Log-rank test showed a gradual decline in survival among high-risk subjects during follow-up period, indicating that patients with higher mortality risk probability are more likely to die (log-rank test *p* < 10^–3^) (Figure 4).

**FIGURE 5:**
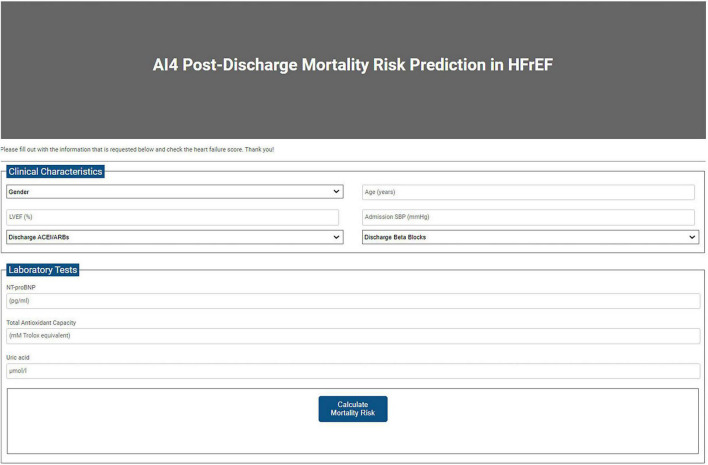
Post-discharge mortality risk calculator.

Based on the GLM model, an online post-discharge mortality risk calculator was created.^1^ This tool calculated the estimated individual probability of dying using clinical characteristics and laboratory tests (Figure 5).

## Discussion

In the present study, we developed a new predictive model for post-discharge mortality among patients admitted for newly diagnosed or exacerbated HFrEF. In addition to clinical patient characteristics and NT-proBNP, 6 oxidative stress markers were considered as candidate variables for risk prediction. Among the three developed models, the GLM presented the best performance for death prediction in HFrEF. Hence, this model was selected to predict mortality risk among our study patients. The predictors included in the GLM model were age, female sex, beta blockers, NT-proBNP levels, LVEF, TAC levels, admission SBP, ACEI/ARBs and UA levels.

To our knowledge, our study presents the first predictive model for post-discharge mortality risk among HFrEF patients, based on a combination of clinical patients’ characteristics, NT-proBNP and oxidative stress markers. Previous mortality risk prediction models among patients with HFrEF incorporated clinical characteristics and NT-proBNP levels (24, 48, 49). The integration of few readily obtainable variables is also a great advantage of our model highlighting the potential use and implementation of this artificial intelligence tool in clinical practice. A number of the predictors identified in our model were also included in the PREDICT-HF models, including age, beta blockers, NT-proBNP levels, LVEF, admission SBP and UA levels; though the former has fewer variables (48).

On the statistical significance level, our model had a good discriminative power of post-discharge death with an AUC of 74.5%, which is comparable to the Seattle HF Model, one of the most extensively used models, achieving an AUC of 72.9% for 1-year survival (16). Likewise, the AUCs from recently developed mortality risk prediction models in HFrEF ranked from 67 to 78% (24, 48–50). Furthermore, our model was developed based on the original statistical approach. The traditional statistical method based on logistic regression, commonly applied in previous predictive models for HFrEF, was explored (51). In addition, two novel machine learning approaches (random forest and extreme gradient boosting) were also applied to predict post-discharge mortality in patients with HFrEF.

Regarding oxidative stress markers, UA and TAC were retained as significant predictors of post-discharge mortality risk in our model. Our analysis showed that high level of plasma UA before discharge was significantly associated with all increased post-discharge death risk among HFrEF patients using univariate and multivariate analysis. Patients with UA levels > 460 μmol/l were at high risk to die during follow up periods. In addition, UA was one of the top five features selected in RF and XGBoost models. Several clinical studies showed that elevated level of UA was an important risk factor of mortality in HFrEF (52, 53). Further, UA has been incorporated in a clinically validated model to predict mortality in HFrEF, displaying independent predictive ability in the Seattle Heart Failure Model (16). UA, the final product of purine degradation, is one of the major endogenous antioxidants in the human plasma (54, 55). As a putative protective mechanism, increased levels of plasma UA may be a compensatory mechanism to limit the damage of inappropriate ROS production (56). Xanthine oxidoreductase (XO) is the enzyme that catalyzes the conversion of hypothanthine to UA in the final steps of purine catabolism (57). Elevated UA levels reflect the amplified activity of XO, a key enzyme in the production of ROS (58). Elevated UA levels reflect the amplified activity of XO, a key enzyme in the production of ROS (28). Accordingly, previous studies reported that an increased level of UA was associated with disease severity and correlated positively with left ventricular remodeling indices in patients with HFrEF (31, 59).

TAC has also emerged as an important prognostic marker of mortality in the GLM model. Our study elucidated that an elevated level of plasma TAC was an independent predictor for post-discharge mortality even after complete adjustment, including sex, age, BMI, NT-proBNP and significant risk factors. We also introduced the 11.5 mM trolox equivalent cut-off of plasma TAC before discharge as a novel tool for risk stratification. Previous studies reported an association between high levels of TAC and death risk in HFrEF (60) and urgent heart transplantation among patients with non-ischemic cardiomyopathy (36). Similar predictive significance of the TAC level was also found in other cardiovascular diseases such as coronary artery disease (61). Indeed, Tomandlova et al. showed that TAC level was significantly higher in patients with more severe coronary artery disease and worse prognosis. In addition, previous studies showed an association between high TAC level and mortality among patients with ischemic stroke and severe septic (62, 63). TAC is an integrated parameter rather than a simple sum of measurable antioxidants, representing the cumulative action of all plasma antioxidants (64). A higher level of TAC may reflect a greater antioxidant response due to intensified production of ROS. It has also been suggested that elevated TAC level in non-survivors patients represents a compensating mechanism of an organism for depleted antioxidative components (65). Overall, the association between increased mortality risk and higher levels of UA and TAC, observed in this study and earlier studies, suggested that these easy and accessible markers of oxidative stress could be valuable biomarkers and prognostic factors in patients with HFrEF.

Our multivariable model confirmed the strong predictive value of NT-proBNP. Results obtained in this study showed that an elevated level of plasma NT-proBNP was a strong predictor of post-discharge mortality risk in both univariate and multivariate analysis. Previous clinical studies have shown that NT-proBNP level was significantly associated with increased mortality risk in HFrEF (24, 66). Moreover, the American Heart Association/American College of Cardiology HF guidelines have recommended measuring Natriuretic peptide biomarkers for prognostication among patients with HFrEF (20). Indeed, the Natriuretic peptide tests are still underutilized in different Tunisian centers as in many African countries which could implement this test in routine clinical practice (5). In the present study, we present the first prospective evaluation of NT-proBNP levels among Tunisian patients with HFrEF. In this context, our study confirms the predictive value of this test and encourages its implementation in routine clinical practice in Africa.

In addition, the clinical variables identified in our model include age, female sex, lower admission SBP and lower LVEF. These variables were established as prognostic markers in HFrEF (17, 18). Beta blockers and ACEI/ARBs were also retained in the model. Indeed, our findings reported a lower risk of post-discharge mortality in patients taking these medications before discharge, which are similar to those observed in several reports (67, 68). The protective role of these drugs impacting mortality risk among HFrEF patients may be partially due to their potent antioxidant properties (69, 70), indicating the important prognostic role of oxidative stress in patients with HFrEF.

Using the GLM model, we created a simple calculator allowing the identification of patients with heightened post-discharge death risk. Regarding its practical application, this calculator is promising to be applied in future clinical practice. Indeed, this easy-to-use calculator can be easily implemented in clinical practice. It is anticipated to aid physicians to calculate the estimated mortality risk. Indeed, the identification of patients at heightened post-discharge death risk can be used to alter care, with closer follow-up and potential earlier consideration of advanced therapies. Accurate estimation of mortality risk in patients with HFrEF may allow clinicians and patients to make important decisions regarding the appropriateness and timing of disease-modifying treatments and advanced therapies (48). In addition, identifying factors common to patients at high risk of post-discharge mortality may reveal potential targets for interventions to improve prognosis. The implementation of these risk prediction tools is relevant to healthcare, particularly in the clinical decision-making (71). Shared decision making can improve motivation for therapy adherence and lifestyle change. Furthermore, the application of these prognosis tools can guide the allocation of healthcare resources and reduce costs (71).

## Limitations

We present a single center study conducted in Hedi Chaker Hospital of Sfax presenting a relatively small number of patients and regional limitations. However, this sample size had sufficient statistical power to detect mean differences. At the clinical level, our study was restricted to HFrEF patients due to its high prevalence in Tunisia (8). Then, this model may not be generalizable to HFpEF patients and HFrEF patients with major life-altering comorbidities including, acute myocardial infarction, severe valvular disease requiring surgery and renal failure requiring dialysis. We also precise that data regarding the use of devices, such as implantable cardiac defibrillator and cardiac resynchronization therapy, were not available. However, the inclusion of such variables may add prognostic power to our model. At statistical level, we consider that the absence of external validation represents an acknowledged limitation, which was circumvented by good discrimination in internal validation of our model.

## Future directions

Our pilot study should be expanded to different medical centers in order to include patients from different parts of Tunisia. We also encourage further validation of our risk model in other populations of HFrEF patients. Larger future prospective multicenter studies with larger numbers of patients are needed to confirm the predictive value of oxidative stress markers, assess for cost effectiveness and to define the implications for earlier interventions to improve prognosis.

## Conclusion

In conclusion, we developed a new predictive model for post-discharge all-cause death in patients with HFrEF based on a combination of clinical patient characteristics, NT-proBNP and oxidative stress markers. This new model assisted by a simple-to-use calculator may allow the identification of patients at heightened post-discharge mortality risk and could assist the treatment decision-making.

## Data availability statement

The original contributions presented in this study are included in the article/Supplementary material, further inquiries can be directed to the corresponding author.

## Ethics statement

This study was approved by the Local Ethics Committee of CHU Hédi Chaker of Sfax (Tunisia), in accordance with the principles expressed in the Declaration of Helsinki (CPP Sud 0276/2017). The patients/participants provided their written informed consent to participate in this study.

## Author contributions

NK contributed to the conceptualization and methodology. IG, SC, FZ, and LA made substantial contributions to samples and data acquisition. RA and WO contributed to data analysis. IG contributed to data interpretation and drafting of the manuscript. IG, FB, WO, NK, and AR reviewed and edited the manuscript. All authors had full access to all the data in the study and agreed to the published version of the manuscript.

## Footnotes

1
https://heartcheckapp.herokuapp.com/
