# Supplementary material for: Oxidative stress markers-driven prognostic model to predict post-discharge mortality in heart failure with reduced ejection fraction
Source: Front Cardiovasc Med. 2022 Nov 7;9:1017673. doi: 10.3389/fcvm.2022.1017673 (PMC9676261; doi:10.3389/fcvm.2022.1017673)
Supplement: Supplementary file 1 [file Data_Sheet_1.docx]

Supplementary Material

**Scripts and algorithms used in R**

**# Kaplan meier survival analysis**

setwd("D:/Travail imen Gthif")

KaplanMaierr <-read.csv2("KaplanMaier.csv",header=TRUE,sep=";",dec=",",na.strings = "")

library(survival)

library (survminer, quietly = TRUE)

km_global <- survfit(Surv(time, status) ~ 1, data = KaplanMaierr)

ggsurvplot(km_global, pval = TRUE,conf.int =FALSE,xlab = "Days",xlab = "Days", ylab = "survival probability")

ggsurvplot(fit = survfit(Surv(time, status) ~ 1, data = KaplanMaierr),conf.int =FALSE, xlab = "Days", ylab = "Overall survival probability")

km_UA <- survfit(Surv(Days, Status) ~ UA, data = KaplanMaierr)

ggsurvplot(fit =km_UA,conf.int =FALSE,pval = TRUE, xlab = "Days", ylab = "Survival probability")

km_NT_proBNP <- survfit(Surv(Days, Status) ~ NT_proBNP, data = KaplanMaierr)

ggsurvplot(fit =km_NT_proBNP,conf.int =FALSE,pval = TRUE, xlab = "Days", ylab = "Survival probability")

km_TAC <- survfit(Surv(Days, Status) ~ TAC, data = KaplanMaierr)

ggsurvplot(fit =km_TAC,conf.int =FALSE,pval = TRUE, xlab = "Days", ylab = "Survival probability")

km_score <- survfit(Surv(Days, Status) ~ Score, data = KaplanMaierr)

ggsurvplot(fit =km_score,conf.int =FALSE,pval = TRUE, xlab = "Days", ylab = "Survival probability")

**# Multivariable Cox regression**

Cox_data=read.csv2("Cox_mortality.csv",header=TRUE,sep=";",dec=",",na.strings = "")

mod1 <- coxph(Surv(Time, Status) ~ .,data = Cox_data)

mod2 <- stepAIC(mod1,method = c("both","backward", "forward"))

summary(mod2)

cbind(exp(mod2$coef),exp(confint(mod2)))

library(ROCR)

library(blorr)

library(ROCR)

library(blorr)

library(risksetROC)

library(survminer)

library(survival)

ggforest(mod2,data=Cox_data)

test <- cox.zph(mod2)

**# Binary logistic regression**

setwd("D:/Travail imen Gthif/”)

GLM=read.csv2("GLM.csv",header=TRUE,sep=";",dec=",",na.strings = "")

model_complet=glm(Status~.,data=GLM,family=binomial)

require (MASS)

model_complet.step=step(model_complet, direction="backward")

summary(model_complet.step)

cbind(exp(model_complet.step$coef),exp(confint(model_complet.step)))

model1=stepAIC(model_complet, direction = c("both", "backward", "forward"))

summary(model_complet.step)

cbind(exp(model_complet.step$coef),exp(confint(model_complet.step)))

**Scripts and algorithms used in phyton**

# -*- coding: utf-8 -*-

"""Interface - Mortality Heart Disease.ipynb

Automatically generated by Colaboratory.

Original file is located at

https://colab.research.google.com/drive/1CO3oLY_ZsVq-iBlCQXYxBuppEPkr1Asn

"""

import pandas as pd

df=pd.read_excel('/content/dataset.xlsx')

df.head()

def df_to_plotly(df):

return {'z': df.values.tolist(),

'x': df.columns.tolist(),

'y': df.index.tolist() }

import plotly.graph_objects as go

X=df.drop(' Mortality ',axis=1)

dfNew = X.corr()

fig = go.Figure(data=go.Heatmap(df_to_plotly(dfNew)))

fig.show()

**"""# **Features Importance****

**# **Random Forest****

**"""**

from sklearn import preprocessing

from sklearn.model_selection import train_test_split

from sklearn.ensemble import RandomForestRegressor

X=df.drop('Mortality ',axis=1)

Y=df['Mortality ']

X_train, X_test, Y_train, Y_test = train_test_split(X, Y, test_size = 0.2)

# Build the model with the random forest regression algorithm:

model = RandomForestRegressor(max_depth=6, random_state=0, n_estimators=10)

model.fit(X_train, Y_train)

!pip install shap

# Commented out IPython magic to ensure Python compatibility.

import shap

# %matplotlib inline

shap_values = shap.TreeExplainer(model).shap_values(X_train)

shap.summary_plot(shap_values, X_train, plot_type="bar")

import matplotlib.pyplot as plt

f = plt.figure()

shap.summary_plot(rf_shap_values, X_train)

f.savefig("/summary_plot1.png", bbox_inches='tight', dpi=600)

# Initialize your Jupyter notebook with initjs(), otherwise you will get an error message.

shap.initjs()

# Write in a function

def shap_plot(j):

explainerModel = shap.TreeExplainer(model)

shap_values_Model = explainerModel.shap_values(S)

p = shap.force_plot(explainerModel.expected_value, shap_values_Model[j], S.iloc[[j]])

return(p)

import numpy as np

# Get the predictions and put them with the test data.

X_output = X_test.copy()

X_output.loc[:,'predict'] = np.round(model.predict(X_output))

# Randomly pick some observations

random_picks = np.arange(0,24,2) # Every 50 rows

S = X_output.iloc[random_picks]

S

shap.initjs()

shap_plot(0)

shap.initjs()

shap_plot(1)

shap.initjs()

shap_plot(3)

**"""# **XGboost**"""**

!pip install xgboost==1.0.1

from sklearn import preprocessing

from sklearn.model_selection import train_test_split

from xgboost import XGBRegressor

X=df.drop('Mortality ',axis=1)

Y=df['Mortality ']

X_train, X_test, Y_train, Y_test = train_test_split(X, Y, test_size = 0.2)

# Build the model with the random forest regression algorithm:

model = XGBRegressor()

model.fit(X_train, Y_train)

!pip install shap

# Commented out IPython magic to ensure Python compatibility.

import shap

# %matplotlib inline

shap_values = shap.TreeExplainer(model).shap_values(X_train)

shap.summary_plot(shap_values, X_train, plot_type="bar")

import matplotlib.pyplot as plt

f = plt.figure()

shap.summary_plot(shap_values, X_train)

f.savefig("/summary_plot1.png", bbox_inches='tight', dpi=600)

# Write in a function

def shap_plot(j):

explainerModel = shap.TreeExplainer(model)

shap_values_Model = explainerModel.shap_values(S)

p = shap.force_plot(explainerModel.expected_value, shap_values_Model[j], S.iloc[[j]])

return(p)

# Using a random sample of the dataframe for better time computation

X_sampled = X_train.sample(50, random_state=10)

# explain the model's predictions using SHAP values

# (same syntax works for LightGBM, CatBoost, and scikit-learn models)

explainer = shap.TreeExplainer(model)

shap_values = explainer.shap_values(X_sampled)

# Initialize your Jupyter notebook with initjs(), otherwise you will get an error message.

shap.initjs()

# visualize the first prediction's explanation

shap.force_plot(explainer.expected_value, shap_values[0,:], X_sampled.iloc[0,:])

# Initialize your Jupyter notebook with initjs(), otherwise you will get an error message.

shap.initjs()

# visualize the first prediction's explanation

shap.force_plot(explainer.expected_value, shap_values[1,:], X_sampled.iloc[1,:])

# Initialize your Jupyter notebook with initjs(), otherwise you will get an error message.

shap.initjs()

# visualize the first prediction's explanation

shap.force_plot(explainer.expected_value, shap_values[2,:], X_sampled.iloc[2,:])

shap.initjs()

# visualize the training set predictions

shap.force_plot(explainer.expected_value, shap_values, X_train)

**# -*- coding: utf-8 -*-**

**"""Leave One Out Classification (LOOC).ipynb**

Automatically generated by Colaboratory.

Original file is located at

https://colab.research.google.com/drive/15nGcwJP8csgTEz__bGEV0jVHWkTmaoD9

**### **Random Forest Leave One Out Classification****

**"""**

import pandas as pd

df=pd.read_excel('/content/Interface.xlsx')

X=df.drop('Mortality ',axis=1)

y=df['Mortality ']

# loocv to automatically evaluate the performance of a random forest classifier

from numpy import mean

from numpy import std

from sklearn.datasets import make_blobs

from sklearn.model_selection import LeaveOneOut

from sklearn.model_selection import cross_val_score, cross_val_predict

from sklearn.ensemble import RandomForestClassifier

# create dataset

#X, y = make_blobs(n_samples=100, random_state=1)

# create loocv procedure

cv = LeaveOneOut()

# create model

model = RandomForestClassifier(random_state=1)

# evaluate model

scoresrf = cross_val_score(model, X, y, scoring='accuracy', cv=cv, n_jobs=-1)

y_predrf = cross_val_predict(model, X, y, cv=cv)

# report perfofrom sklearn import datasets

print('Accuracy: %.3f Standard Deviation:(%.3f)' % (mean(scoresrf), std(scoresrf)))

from sklearn.metrics import matthews_corrcoef

mcc_rf=matthews_corrcoef(y, y_predrf)

print(mcc_rf)

import numpy as np

from sklearn import metrics

fpr, tpr, thresholds = metrics.roc_curve(y, y_predrf)

auc_rf=metrics.auc(fpr, tpr)

print(auc_rf)

from sklearn.metrics import confusion_matrix, classification_report

cr = classification_report(y, y_predrf)

print(cr)

**"""### **Linear Logistic Leave One Out Classification**"""**

import pandas as pd

df=pd.read_excel('Interface.xlsx')

X=df.drop('Mortality ',axis=1)

y=df['Mortality ']

# loocv to automatically evaluate the performance of a random forest classifier

from numpy import mean

from numpy import std

from sklearn.datasets import make_blobs

from sklearn.model_selection import LeaveOneOut

from sklearn.model_selection import cross_val_score, cross_val_predict

from sklearn.linear_model import LogisticRegression

from sklearn.pipeline import make_pipeline

from sklearn.preprocessing import StandardScaler

# create dataset

#X, y = make_blobs(n_samples=100, random_state=1)

# create loocv procedure

cv = LeaveOneOut()

# create model

pipe = make_pipeline(StandardScaler(), LogisticRegression())# evaluate model

scoreslr = cross_val_score(pipe, X, y, scoring='accuracy', cv=cv, n_jobs=-1)

y_predlr = cross_val_predict(pipe, X, y, cv=cv)

# report perfofrom sklearn import datasets

print('Accuracy: %.3f Standard Deviation:(%.3f)' % (mean(scoreslr), std(scoreslr)))

from sklearn.metrics import matthews_corrcoef

mcc_lr=matthews_corrcoef(y, y_predlr)

print(mcc_lr)

import numpy as np

from sklearn import metrics

fpr, tpr, thresholds = metrics.roc_curve(y, y_predlr)

auc_lr=metrics.auc(fpr, tpr)

print(auc_lr)

import pickle

pickle.dump(pipe, open('model.pkl', 'wb'))

#Import needed libraries

from sklearn.metrics import confusion_matrix, classification_report

import seaborn as sns

cr = classification_report(y, y_predlr)

cm = confusion_matrix(y, y_predlr)

tn, fp, fn, tp = confusion_matrix(y, y_predlr).ravel()

cm = [[tp,fp],[fn,tn]]

#Plot the matrix

sns.heatmap(cm, annot=True, fmt = "d", cmap="Spectral")

print(y_predlr)

cr = classification_report(y, y_predlr)

print(cr)

**"""### **XGboost Leave One Out Classification**"""**

import pandas as pd

df=pd.read_excel('/content/Interface.xlsx')

X=df.drop('Mortality ',axis=1)

y=df['Mortality ']

# loocv to automatically evaluate the performance of a random forest classifier

from numpy import mean

from numpy import std

from sklearn.datasets import make_blobs

from sklearn.model_selection import LeaveOneOut

from sklearn.model_selection import cross_val_score, cross_val_predict

from xgboost import XGBClassifier

# create dataset

#X, y = make_blobs(n_samples=100, random_state=1)

# create loocv procedure

cv = LeaveOneOut()

# create model

model = XGBClassifier(random_state=1)

# evaluate model

scoresxg = cross_val_score(model, X, y, scoring='accuracy', cv=cv, n_jobs=-1)

y_predxg = cross_val_predict(model, X, y, cv=cv)

# report perfofrom sklearn import datasets

print('Accuracy: %.3f Standard Deviation:(%.3f)' % (mean(scoresxg), std(scoresxg)))

from sklearn.metrics import matthews_corrcoef

mcc_xg=matthews_corrcoef(y, y_predxg)

print(mcc_xg)

import numpy as np

from sklearn import metrics

fpr, tpr, thresholds = metrics.roc_curve(y, y_predxg)

auc_xg=metrics.auc(fpr, tpr)

print(auc_xg)

cr = classification_report(y, y_predxg)

print(cr)

data = {'Machine Learning': ['Random Forest', 'Linear Logistic', 'XGBoost'], 'Accuracy': [mean(scoresrf)*100, mean(scoreslr)*100, mean(scoresxg)*100], 'Standard Devation': [mean(scoresrf), mean(scoreslr), mean(scoresxg)], 'MCC': [mcc_rf, mcc_lr, mcc_xg], 'AUC': [auc_rf, auc_lr, auc_xg]}

# Create DataFrame

df = pd.DataFrame(data)

# Print the output.

df.head()
